# Focal or Macro-reentrant (Dual-loop) Atrial Tachycardia? The Role of the Ligament of Marshall

**DOI:** 10.19102/icrm.2021.120116S

**Published:** 2021-01-15

**Authors:** Francesco Peruzza, Carlo Angheben, Massimiliano Maines, Paolo Moggio, Domenico Catanzariti, Camilla Bonvicini, Stefano Indiani, Maurizio Del Greco

**Affiliations:** ^1^Santa Maria del Carmine Hospital, Rovereto, TN, Italy; ^2^Abbott, Milan, Italy

**Keywords:** Arrhythmogenic right ventricular cardiomyopathy, atrial tachycardia, left atrial appendage, ligament of Marshall

A 56-year-old man was referred to our institution for cardiac ablation because of several episodes of palpitations and thoracic pain with an electrocardiogram suggestive for left atrial tachycardia (160 bpm). He was affected by arrhythmogenic right ventricular cardiomyopathy (ARVC) with left ventricular involvement and a plakophillin-2 gene mutation. A cardiac magnetic resonance imaging scan showed biventricular fibro-fatty infiltration with matching late gadolinium enhancement.

An electrophysiological study was performed in combination with electroanatomic mapping (EnSite Precision™). An activation map of the left atrium during tachycardia was created with the Advisor™ HD Grid Mapping Catheter, Sensor Enabled™. The region of earliest activation was identified at the base of the left atrial appendage (LAA), confirmed also by the EnSite™ LiveView Dynamic Display. The activation wavefront was suggestive for a focal-origin tachycardia from the LAA with an uncommon line of block between the LAA and left superior pulmonary vein along the left atrial roof **(Figure 1)**. However, we could not exclude a macro-reentrant (dual-loop) tachycardia **(Video 1)** with a slow-conduction isthmus inside the vein of Marshall with its fibrous and muscular component (Marshall bundle) as supported by the presence of fragmented potential along the line of block.

Ablation with 25 W (TactiCath™ Contact Force Ablation Catheter, Sensor Enabled™) at the earliest activation site terminated the tachycardia within three seconds. At six months of follow-up, no recurrence of the arrhythmia was observed.

## Figures and Tables

**Figure 1:**
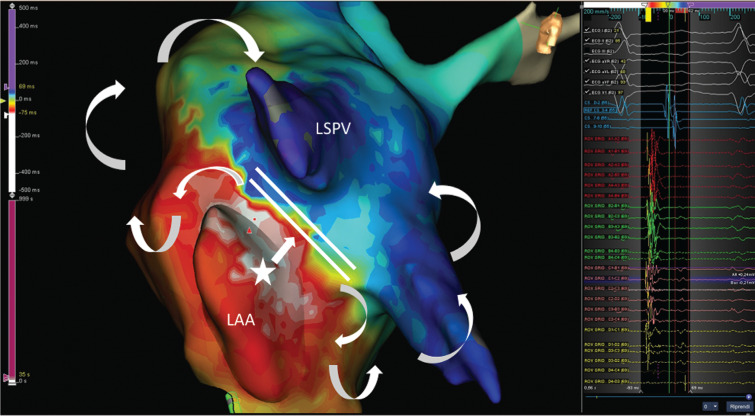
Left atrium activation mapping with the Advisor™ HD Grid Catheter. The activation wavefront was suggestive for a focal origin of tachycardia from the LAA with an uncommon line of block between the LAA and left superior pulmonary vein along the left atrial roof. LAA: left atrial appendage; LSPV: left superior pulmonary vein.

**Video 1.** Left atrium activation mapping with the Advisor™ HD Grid catheter. The activation wavefront was suggestive for a focal origin of tachycardia from the LAA with an uncommon line of block between the LAA and left superior pulmonary vein along the left atrial roof. However, we could not exclude a macro-reentrant (dual-loop) tachycardia with a slow conduction isthmus inside the vein of Marshall.

